# Supplementary material for: In vitro generation of human pluripotent stem cell derived lung organoids
Source: eLife. 2015 Mar 24;4:e05098. doi: 10.7554/eLife.05098 (PMC4370217; doi:10.7554/eLife.05098)
Supplement: Supplementary file 1. — Publicly available RNAseq datasets for human fetal lung representing a range of gestational stages and for adult human lung. DOI: http://dx.doi.org/10.7554/eLife.05098.025 [file elife05098s001.docx]

| **Table 1** | | | | |
| --- | --- | --- | --- | --- |
| **Sample Label** | **Description** | **Source** | **Donor ID** | **Accession #** |
| Lung_A_1 | Adult Lung 3e | EMBL-EBI ArrayExpress | V80 | E-MTAB-1733 |
| Lung_A_2 | Adult Lung 3f | EMBL-EBI ArrayExpress | V81 | E-MTAB-1733 |
| Lung_A_3 | Adult Lung 4a | EMBL-EBI ArrayExpress | V130 | E-MTAB-1733 |
| Lung_A_4 | Adult Lung 4b | EMBL-EBI ArrayExpress | V131 | E-MTAB-1733 |
| Lung_A_5 | Adult Lung 4d | EMBL-EBI ArrayExpress | V133 | E-MTAB-1733 |
| Lung_A_6 | Fetal day 105, lung | GEO Datasets | H-24005 | GSM1101693 |
| Lung_F_2 | Fetal day 105, lung | GEO Datasets | H-24111 | GSM1101708 |
| Lung_F_3 | Fetal day 108, lung | GEO Datasets | H-23887 | GSM1101684 |
| Lung_F_4 | Fetal day 91, lung | GEO Datasets | H-23914 | GSM1101685 |
| Lung_F_5 | Fetal day 96, lung | GEO Datasets | H-24089 | GSM1101699 |
| Lung_F_6 | Fetal day 98, lung | GEO Datasets | H-23964 | GSM1101687 |
