# Supplementary material for: In vitro generation of human pluripotent stem cell derived lung organoids
Source: eLife. 2015 Mar 24;4:e05098. doi: 10.7554/eLife.05098 (PMC4370217; doi:10.7554/eLife.05098)
Supplement: Supplementary file 2. — Antibody information and dilutions. DOI: http://dx.doi.org/10.7554/eLife.05098.026 [file elife05098s002.docx]

| **TABLE 2** | | | | |
| --- | --- | --- | --- | --- |
| **Primary Antibody** | **Source** | **Catalog #** | **Dilution** | **Clone** |
| Chicken anti-GFP | Abcam | Ab13970 | 1:500 | polyclonal |
| Goat anti-β-Catenin (βCAT) | Santa Cruz Biotechnology | sc-1496 | 1:200 | C-18 |
| Goat anti-CC10 | Santa Cruz Biotechnology | sc-9770 | 1:200 | C-20 |
| Goat anti-E-Cadherin (ECAD) | R&D Systems | AF748 | 1:100 | N-19 |
| Goat anti-FOXA2 | Santa Cruz Biotechnology | sc-6554 | 1:100 | M-20 |
| Goat anti-SOX2 | Santa Cruz Biotechnology | sc-17320 | 1:100 | Y-17 |
| Goat anti-SOX9 | R&D Systems | AF3075 | 1:500 | polyclonal |
| Goat anti-SOX17 | R&D Systems | AF1924 | 1:500 | polyclonal |
| Goat anti-VIMENTIN (VIM) | Santa Cruz Biotechnology | sc-7558 | 1:100 | S-20 |
| Mouse anti-Acetylated Tubulin (ACTTUB) | Sigma-Aldrich | T7451 | 1:1000 | 6-11B-1 |
| Mouse anti-E-Cadherin (ECAD) | BD Transduction Laboratories | 610181 | 1:500 | 36/E-Cadherin |
| Mouse anti-FOXJ1 | eBioscience | 14-9965-82 | 1:500 | 2A5 |
| Rabbit anti-FOXA2 | Seven Hills Bioreagents | WRAB-FOXA2 | 1:1000 | aa7-86 |
| Rabbit anti-NKX2.1 | Abcam | ab76013 | 1:200 | EP1584Y |
| Rabbit anti-P63 | Santa Cruz Biotechnology | sc-8344 | 1:200 | H-129 |
| Rabbit anti-PAX8 | Proteintech Group | 10336-1-AP | 1:500 | Ag0306 |
| Rabbit anti-PDPN | Santa Cruz Biotechnology | sc-134482 | 1:200 | FL-162 |
| Rabbit anti-N-Terminal Pro SP-C (SFTPC) | Seven Hills Bioreagents | WRAB-9337 | 1:200 | aa1-35 |
| Rabbit anti-SOX2 | Seven Hills Bioreagents | WRAB-SOX2 | 1:500 | polyclonal |
| Cy3- Mouse anti Actin-alpha smooth muscle (SMA)* | Sigma | C6198 | 1:400 | MonoClonal |
| Rabbit anti-Sufactant Protein B (SFTPB) | Santa Cruz Biotechnology | sc-13978 | 1:200 | H-300 |
| **Secondary Antibody** | **Source** | **Catalog #** | **Dilution** |  |
| Donkey anti-goat 488 | Jackson Immuno | 705-545-147 | 1:500 |  |
| Donkey anti-goat 647 | Jackson Immuno | 705-605-147 | 1:500 |  |
| Donkey anti-goat Cy3 | Jackson Immuno | 705-165-147 | 1:500 |  |
| Donkey anti-mouse 488 | Jackson Immuno | 715-545-150 | 1:500 |  |
| Donkey anti-mouse 647 | Jackson Immuno | 415-605-350 | 1:500 |  |
| Donkey anti-mouse Cy3 | Jackson Immuno | 715-165-150 | 1:500 |  |
| Donkey anti-rabbit 488 | Jackson Immuno | 711-545-152 | 1:500 |  |
| Donkey anti-rabbit 647 | Jackson Immuno | 711-605-152 | 1:500 |  |
| Donkey anti-rabbit Cy3 | Jackson Immuno | 711-165-102 | 1:500 |  |
| Donkey anti-goat 488 | Jackson Immuno | 705-545-147 | 1:500 |  |
| Donkey anti-goat 647 | Jackson Immuno | 705-605-147 | 1:500 |  |
| Donkey anti-goat Cy3 | Jackson Immuno | 705-165-147 | 1:500 |  |
| Donkey anti-mouse 488 | Jackson Immuno | 715-545-150 | 1:500 |  |
| Donkey anti-mouse 647 | Jackson Immuno | 415-605-350 | 1:500 |  |
| Donkey anti-mouse Cy3 | Jackson Immuno | 715-165-150 | 1:500 |  |
| Donkey anti-rabbit 488 | Jackson Immuno | 711-545-152 | 1:500 |  |
| Donkey anti-rabbit 647 | Jackson Immuno | 711-605-152 | 1:500 |  |
| Donkey anti-rabbit Cy3 | Jackson Immuno | 711-165-102 | 1:500 |  |

***Secondary antibody conjugated to the primary antibody**
