# Supplementary material for: In vitro generation of human pluripotent stem cell derived lung organoids
Source: eLife. 2015 Mar 24;4:e05098. doi: 10.7554/eLife.05098 (PMC4370217; doi:10.7554/eLife.05098)
Supplement: Supplementary file 3. — Primer sequences. DOI: http://dx.doi.org/10.7554/eLife.05098.027 [file elife05098s003.docx]

| **TABLE 3** | | |
| --- | --- | --- |
| **Primer Name** | **Forward Sequence** | **Reverse Sequence** |
| *CDX2* | GGGCTCTCTGAGAGGCAGGT | GGTGACGGTGGGGTTTAGCA |
| *ECADHERIN* | TTGACGCCGAGAGCTACAC | GACCGGTGCAATCTTCAAA |
| *FOXA2* | CGACTGGAGCAGCTACTATGC | TACGTGTTCATGCCGTTCAT |
| *FOXJ1* | CAACTTCTGCTACTTCCGCC | CGAGGCACTTTGATGAAGC |
| *HHEX* | CCTCTGTACCCCTTCCCG | GGGGCTCCAGAGTAGAGGTT |
| *HOPX* | GCCTTTCCGAGGAGGAGAC | TCTGTGACGGATCTGCACTC |
| *ID2* | GACAGCAAAGCACTGTGTGG | TCAGCACTTAAAAGATTCCGTG |
| *MUC5AC** | GCACCAACGACAGGAAGGATGAG | CACGTTCCAGAGCCGGACAT |
| *NKX2.1* | CTCATGTTCATGCCGCTC | GACACCATGAGGAACAGCG |
| *NMYC* | CACAGTGACCACGTCGATTT | CACAAGGCCCTCAGTACCTC |
| *P63* | CCACAGTACACGAACCTGGG | CCGTTCTGAATCTGCTGGTCC |
| *PAX8* | TGCCTCACAACTCCATCAGA | CAGGTCTACGATGCGCTG |
| *PDPN* | ACATCCTTTGTTTTTGCCCA | AGTGTCATCTTCTGGCTGGC |
| *PDX1* | CGTCCGCTTGTTCTCCTC | CCTTTCCCATGGATGAAGTC |
| *SCGB1A1* | ATGAAACTCGCTGTCACCCT | GTTTCGATGACACGCTGAAA |
| *SFTPC* | AGCAAAGAGGTCCTGATGGA | CGATAAGAAGGCGTTTCAGG |
| *SOX2* | GCTTAGCCTCGTCGATGAAC | AACCCCAAGATGCACAACTC |
| *SOX9* | GTACCCGCACTTGCACAAC | GTGGtCCTTCTTGTGCTGC |
| *VIMENTIN* | CTTCAGAGAGAGGAAGCCGA | ATTCCACTTTGCGTTCAAGG |

Note: All above primer sequences were obtained from <http://primerdepot.nci.nih.gov/> and all annealing temperatures 55°C unless stated otherwise.

**MUC5AC* Huang, SX *et al.* Efficient generation of lung and airway epithelial cells from human pluripotent stem cells. *Nature Biotechnol*. 1–11 (2013). doi:10.1038/nbt.2754

Annealing temperature 60°C
